# Teaching Fido New ModiFICation Tricks

**DOI:** 10.1371/journal.ppat.1004349

**Published:** 2014-09-25

**Authors:** Jonathan W. Cruz, Nancy A. Woychik

**Affiliations:** Department of Biochemistry and Molecular Biology, Rutgers University, Robert Wood Johnson Medical School, Piscataway, New Jersey, United States of America; University of North Carolina at Chapel Hill School of Medicine, United States of America

The Fic (filamentation induced by cAMP) family of proteins comprises several thousand members that are found in all domains of life [1], [2]. In bacteria, these proteins are often expressed by pathogens as virulence factors that disrupt signaling in the mammalian host cell through inhibition of one or more GTPases or kinases involved in cell signaling. By integrating surprising new insights, this Pearl highlights the spectrum of potent posttranslational modifications catalyzed by the Fic family of proteins in bacterial pathogens. We will also discuss how Fic proteins are predicted to endow bacterial pathogens with the ability to perturb essential signaling pathways in their host cells (for secreted Fic proteins) or enable the pathogen to endure stresses such as antibiotic exposure (for intracellular Fic proteins derived from toxin-antitoxin modules).

All Fic family proteins (also referred to as “Fido” because the family includes Doc toxins [3]) contain a Fic domain of 100–140 amino acids that exhibits two hallmarks, striking structural similarity and a highly conserved, nine amino acid active site (Figure 1). At the structural level, Fic domains have an α-helical core consisting of six or eight α-helices (reviewed in [3], [4]). Yet, there is no significant sequence similarity among all Fic domain proteins beyond the 9-residue active site, the Fic motif. These conserved features alone did not provide clues to the function of Fic domain proteins. Only upon molecular analysis of the *Vibrio parahemolyticus* virulence factor VopS by the Orth group [5] was a precise enzymatic activity of Fic domain proteins revealed. This discovery set the stage for a series of unanticipated twists demonstrating that Fic domains can influence pathogenesis by inactivating target proteins involved in cell signaling by one of four posttranslational modifications: AMP, UMP, phosphocholine, or phosphate (summarized in Figure 2).

**Figure 1:**
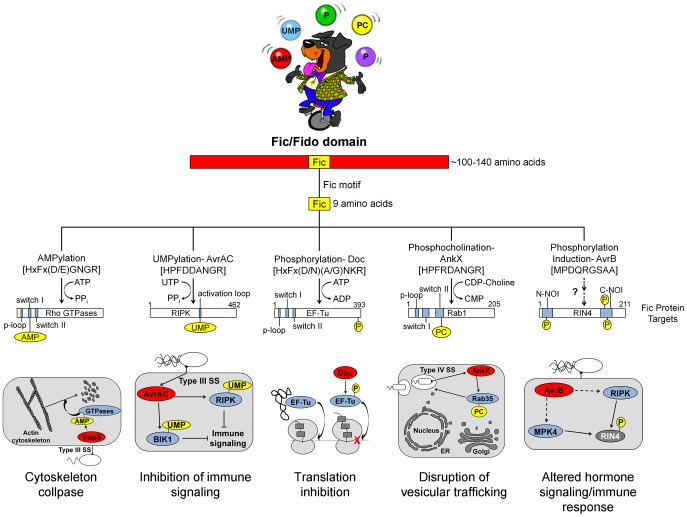
Overview of Fic domain proteins, their targets, and their roles in bacterial virulence. The Fic domain is defined by a conserved structural fold composed of six to eight α-helices (red bar). A nine amino acid Fic motif (yellow box) within this core fold comprises the key residues for catalysis. Both the structure of the Fic domain and the sequence of the Fic motif informs the activity of the Fic domain protein. Important domains of the target proteins are shown as blue bars; modifications as yellow circles (not to scale). Known physiological manifestations of each class of Fic domain protein are illustrated below their target. The exact role of the NOI domains of RIN4 is not known, though they are exclusively represented in plant proteins and have been hypothesized to play a role in host response to pathogens [32]. Abbreviations on juggled balls: AMP, adenosine monophosphate; UMP, uridine monophosphate; P, phosphate; PC, phosphocholine.

**Figure 2:**
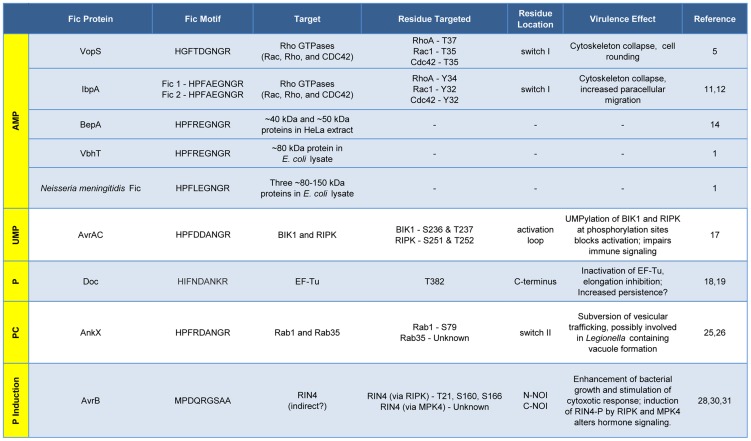
Features and functions of effector proteins that contain a Fic domain. Fic proteins, their targets, and the residue(s) modified are shown. The far left column, running vertically, shows the modification type added by each Fic protein. Dashes, data not known.

## Inactivation of Host Cell GTPases by Addition of AMP

VopS is an effector protein secreted by the foodborne, gastrointestinal pathogen *V. parahemolyticus*.

The Fic domain of VopS catalyzes the stable transfer of a single adenylyl group derived from ATP [5], a process referred to as adenylylation [6] or AMPylation [5], [7]. Although modulation of protein activity by AMPylation had been documented earlier for glutamine synthetase [8], VopS represented the first example of the use of AMPylation in the context of bacterial virulence. The type III secretion system in *V. parahemolyticus* secretes VopS into the mammalian host cell, inactivates Rho family GTPases by AMPylation at a single site in the functionally essential switch I region, and results in collapse of the actin cytoskeleton [5]. VopS-mediated AMPylation of Rho GTPases inhibits NLRC4 inflammasome activation [9] but indirectly activates the Pyrin inflammasome [10]. This counteracting effect of Pyrin stems from its newly identified role as a host cell immune sensor that specifically detects the downstream effects of inactivating modifications to Rho GTPases (i.e., AMPylation, deamidation, glucosylation, and ADP-ribosylation) to restore an inflammatory response [10].

Subsequent work uncovered several more AMPylating Fic domain proteins (Figure 2). IbpA from *Histophilus somni*, an opportunistic pathogen that infects the mucosa of cattle and sheep, has two Fic domains [11]. As with VopS, IbpA AMPylates Rho GTPases at a single site in switch I and leads to cell rounding and cytoskeleton collapse. IbpA is a major virulence factor; *H. somni* strains lacking it are avirulent as defined by their lack of cytotoxicity and the inability to transmigrate across a cultured cell monolayer [12]. Treatment of cultured bovine cells with an antibody to one of the two Fic domains in IbpA also blocks virulence; i.e., it prevents cytotoxicity and cell migration [12]. BepA (*Bartonella* effector protein) from *Bartonella henselae*, which causes cat scratch fever, is one of seven effector proteins translocated by the VirB type IV secretion system [13]. In vitro, BepA AMPylates two proteins in a HeLa cell extract [14]. Another pathogen with a characterized Fic domain protein, *Bartonella schoenbuchensis*, is harbored by a louse fly that typically bites and infects ruminants but may also incidentally infect humans [15]. *B. schoenbuchensis* Fic domain protein VbhT is the toxin component of the VbhA-VbhT toxin-antitoxin (TA) system [1]. VbhT has a carboxy terminal BID (*Bartonella* intracellular delivery) domain that mediates delivery to its mammalian host cell via type IV secretion system [1]. In vitro experiments demonstrated that pure VbhT exhibits AMPylation activity against a single protein in an *Escherichia coli* cell extract [1]. Finally, *Neisseria meningitides*, which most commonly causes meningitis in children and young adults, has a Fic domain protein that appears to AMPylate a few proteins in an *E. coli* cell extract [1].

Distinct from bacterial pathogen virulence factors, there are currently only two eukaryotic Fic domain proteins known to possess AMPylation activity. Humans and *Drosophila* each have a single Fic domain protein. *Drosophila* Fic (also known as CG9523 [3]) plays an essential role in visual neural transmission; flies lacking this protein are viable and fertile, but blind [16]. The human Fic protein, FicD/HYPE, AMPylates Rho GTPases Rac, Rho, and Cdc42 in vitro, but the physiological effects of FicD/HYPE activity are not known [11].

## Inactivation of Kinases by Addition of UMP

There is currently one example of a Fic domain protein that inhibits the activity of its target by covalent addition of a UMP moiety instead of AMP (Figures 1 and 2). AvrAC from the bacterial plant pathogen *Xanthomonas campestris* infects members of the plant family Brassicaceae, including important food crops such as radishes, cauliflower, and cabbage, as well as the model organism *Arabidopsis thaliana*. In *A. thaliana*, AvrAC catalyzes the addition of UMP to the activation loop of kinase targets BIK1 and RIPK, precluding phosphorylation required for their activation [17]. Consequently, the immune response normally mediated by these two kinases is not triggered [17].

## Doc Toxins and Phosphorylation

Although the Doc family of proteins belongs to the Fic/Fido family, they exhibit notable differences in their structural core and catalytic motif. Doc proteins possess only six α-helices in their core folds compared to eight in AMPylating Fic domain proteins [3], and their consensus motif, HxFx(D/N)(A/G)NKR, differs slightly from that found in canonical Fic proteins, HxFx(D/E)GNGR [18], [19] (Figure 1). All Doc family members are toxin components of Phd-Doc TA systems. TA systems are small operons that encode a stable toxin and a labile antitoxin (reviewed in [20]). Under normal conditions, the antitoxin physically interacts with the toxin to inhibit its activity. Specific stress conditions result in the degradation of the antitoxin, freeing the toxin to act on its target, typically leading to bacterial cell growth arrest [20]. In relation to pathogenesis and treatment of bacterial infections, expression of TA systems is associated with an increase in the formation of persister cells [21]. Persister cells exploit slow growth, sometimes to the point of dormancy, to avoid antibiotic-mediated killing and may contribute to the tenacity of certain bacterial pathogens that cause characteristically chronic or recurring infections.

The Doc toxin from the bacteriophage P1 TA system has served as a model for this family and is the only member of this family studied in detail. P1 Doc phosphorylates the essential elongation factor and GTPase EF-Tu, resulting in translation arrest [18], [19]. EF-Tu inactivation through a single phosphorylation event represents a highly effective conduit for regulation of growth and the formation of persister cells.

The slight variations in the conserved Fic motif of Doc, highlighted above, combined with structural differences account for its ability to catalyze a reaction distinct from transfer of an adenylyl group. Canonical Fic proteins possess a glycine at position 8 in their 9-residue Fic motif; instead, Doc family members contain a lysine at this position in the active site. This change forces ATP to bind Doc in an inverted orientation [18], leading to addition of a phosphate moiety to the target protein rather than an adenylyl moiety. Therefore, all Doc family members containing this altered signature motif should also act as kinases. These family members include Doc toxins in the chromosomes of several important pathogens including *Streptococcus pneumoniae*, *Vibrio cholera*, *Clostridium tetani*, and *Salmonella enterica* Typhimurium [22].

## Phosphocholination by AnkX

The Fic domain protein AnkX is a virulence factor from *Legionella pneumophila*, an intracellular pathogen and the causative agent of Legionnaires' disease. Upon inhalation, *L. pneumophila* is taken up by alveolar macrophages and evades killing by altering phagosome–lysosome fusion [23]. The AnkX virulence factor is released into the macrophages by a type IV secretion system [24] and catalyzes the transfer of phosphocholine from CDP-choline to the Rab1 and Rab35 GTPases [25]. Since these GTPases are master regulators of vesicular trafficking, their inactivation results in the disruption of the host cell secretory pathway [26], presumably in a manner that favors establishment and maintenance of *Legionella*-containing vacuoles. Structural studies revealed that AnkX binds to its CDP-choline substrate in the opposite orientation of that for substrates binding canonical Fic proteins, similar to the manner in which Doc binds ATP [27]. This inverted binding is mediated by residues within two distinct domains of AnkX, the Fic domain and a domain unique to AnkX called the CMP domain. AnkX then transfers a phosphocholine moiety onto the target rather than a CMP; however, it does so using the same chemical reaction and catalytic residues as AMPylating Fic proteins [27].

## AvrB and Phosphorylation

The fifth member of the Fic/Fido family is the AvrB effector protein from *Pseudomonas syringae*, a pathogen of plants. This domain has an α-helical core fold that is similar to both Fic and Doc proteins, but contains a drastically different nine amino acid sequence in place of the catalytic Fic motif ([3], Figure 1). After transport into plant cells via a type III secretion system [28], AvrB enhances *P. syringae* growth in susceptible plants [29] and causes cytotoxicity [28]. AvrB interacts with the immune regulator RIN4 and the signaling kinases MPK4 and RIPK; it also induces phosphorylation of RIN4 and MPK4 [28], [30], [31]. However, direct phosphorylation of RIN4 or MPK4 by AvrB was not observed in vitro. Therefore, the mechanism of AvrB-induced phosphorylation is still unclear.

## Common Themes among Bacterial Fic Proteins

This Pearl serves as a snapshot of an evolving field that has uncovered provocative new roles for Fic proteins as modulators of bacterial virulence or persistence. Some notable themes have emerged from family members whose enzymatic activity and cellular targets are known. First, Fic proteins post-translationally modify a functionally critical amino acid through stable, covalent addition of adenosine monophosphate, uridine monophosphate, phosphocholine, or phosphate. With the exception of AvrAC, a single residue in the target protein(s) is modified; AvrAC adds UMP to a contiguous serine-threonine pair [17]. Third, the modification disrupts signaling by, or the function of, the target protein. Fic proteins never enhance the function of their target proteins, in contrast to kinases that are typically activated by modification (phosphorylation). Finally, GTPases are common targets of Fic proteins, a logical choice since they typically have pivotal roles in cell signaling and physiology. In fact, to date, GTPases are the exclusive target of Doc, AnkX, and all of the Fic proteins that AMPylate. With thousands of members of the Fic domain family, this summary undoubtedly represents the tip of the iceberg. Coupled with the complementary body of elegant structural and biophysical studies [4], future advances will surely be accelerated now that a general framework for the function of Fic proteins has been established.
